# Temporal Patterns in Performance of the 30 s Chair-Stand Test Evince Differences in Physical and Mental Characteristics among Community-Dwelling Older Adults in Japan

**DOI:** 10.3390/healthcare8020146

**Published:** 2020-05-28

**Authors:** Akio Goda, Shin Murata, Hideki Nakano, Hina Matsuda, Kana Yokoe, Hodaka Mitsumoto, Kayoko Shiraiwa, Teppei Abiko, Jun Horie

**Affiliations:** Department of Physical Therapy, Faculty of Health Sciences, Kyoto Tachibana University, Kyoto 607-8175, Japan; murata-s@tachibana-u.ac.jp (S.M.); nakano-h@tachibana-u.ac.jp (H.N.); a903017048@tachibana-u.ac.jp (H.M.); a903017058@tachibana-u.ac.jp (K.Y.); a903017050@tachibana-u.ac.jp (H.M.); shiraiwa@tachibana-u.ac.jp (K.S.); abiko@tachibana-u.ac.jp (T.A.); horie-j@tachibana-u.ac.jp (J.H.)

**Keywords:** 30 s chair-stand test, community-dwelling older adults, cognitive function, physical function

## Abstract

Studies involving the 30 s chair-stand test (CS-30) have shown that subjects’ movements can vary during the test, and that these variations may follow several patterns. The present study aimed to define these different patterns and their respective incidences among a population of community-dwelling older adults in Japan. We also investigated, among the patterns identified, potential associations with physical and mental characteristics. The study population comprised 202 community-dwelling older adults. Subjects were classified into four groups based on how their CS-30 performance (defined through sit–stand–sit cycle count) changed over three successive 10 s segments: “steady-goers,” “fluctuators,” “decelerators,” and “accelerators.” Several other measures were also evaluated, including sit-up count, knee-extension strength, toe-grip strength, and Mini-Mental State Examination score. We found that steady-goers and decelerators comprised 70% of the sample. Fluctuators and steady-goers showed comparable physical function. Decelerators exhibited significant correlations between CS-30 score (total cycles) and tasks involving persistence and repetitive actions (*p* < 0.05). In addition, accelerators showed significantly stronger knee extension than steady-goers (*p* < 0.01). Differences in temporal patterns of CS-30 performance corresponded to differences in certain dimensions of physical and mental function. Our findings may be useful for planning and evaluating intervention programs aimed at long-term-care prevention among community-dwelling older adults.

## 1. Introduction

Geriatric preventive care is becoming increasingly important for developed nations with aging populations [1]. Among older adults, several predictors of a future need for long-term care have been identified, including physical factors (e.g., muscular weakness [2], slow walking speed [3], and inability to stand up from a chair [4]) and mental factors (e.g., cognitive decline [5]). Thus, the need for long-term care is not only determined by aging-induced physical deterioration, but is also indirectly influenced by cognitive status [6]. To help lower the risk of requiring nursing care in later years, it is crucial that older adults be assessed holistically, in terms of both physical and mental function, and that steps are taken to maintain and improve their abilities in both domains. However, healthcare professionals work in busy environments and have many responsibilities [7]. Thus, considering the need for efficiency in such work settings, when designing intervention programs for older adults, the ability to characterize physical and mental functioning in simple and uncomplicated terms would be a critical advantage.

The 30 s chair-stand test (CS-30) is widely employed in clinical practice; the test reflects both physical functions (e.g., lower-limb strength [8], core strength [9], muscular endurance [10], balance [11], and mobility [12]) and mental functions (e.g., cognitive skills [13] and attention [14]). Originally devised by Jones et al. [15], Nakatani et al. confirmed the CS-30’s reliability [16] and validity [17] for Japanese samples, and determined reference scores for the Japanese population [16]. Subjects begin the test seated in a chair, and are directed to repeatedly stand up and sit down at maximal effort over a 30 s period. Healthy community-dwelling older adults are expected to perform these “sit–stand–sit” cycles at a steady, rhythmic rate; however, empirical observations have shown that this rhythm actually varies over the duration of the task. Perhaps these deviations follow specific patterns and distributions among community-dwelling older adults, or are influenced by subjects’ physical and mental attributes; however, as few studies have focused on how subjects’ movements vary during CS-30 performance, this remains an open question.

In summary, variations in CS-30 performance—namely, specific patterns by which the sit–stand–sit cycle rate changes over the course of the task—may be more informative than merely considering the number of cycles completed. If so, the predictive power of the CS-30, combined with its ubiquity in geriatric medicine, would make it a highly valuable tool for medical professionals who operate in the field of long-term care prevention. Thus, we hypothesized that different patterns of CS-30 performance evince the physical and mental characteristics of community-dwelling older adults. In this study, we aimed to define the different patterns of CS-30 performance and their respective incidences among a population of community-dwelling Japanese older adults, and to investigate how physical and mental characteristics vary among the identified patterns. 

## 2. Materials and Methods

This study comprises a cross-sectional investigation of 202 community-dwelling older adults (Table 1). Measurements were taken between 9 September and 13 September, 2019. Participants were recruited via informational flyers—which included the statement that there will be no financial compensation for participation—distributed in the city of Yasu in Shiga Prefecture, Japan, between June and August 2019. The inclusion criteria were: (1) aged 60 years or older, (2) no indications of marked cognitive impairment (Mini-Mental State Examination [MMSE] score < 24), and (3) no difficulty in walking independently. Candidates were excluded if they (1) did not understand the directions for the physical or cognitive tests, or (2) had difficulty walking independently. This study was conducted in accordance with the principles of the Declaration of Helsinki. All subjects provided written informed consent; none were cognitively impaired. This study was approved by the ethics committee of Kyoto Tachibana University (approval no. 17-14).

In addition to CS-30 performance as the independent variable, subjects were assessed in terms of several other dimensions, which were the dependent variables. Muscle function was evaluated through sit-up count, knee-extension strength, and toe-grip strength. Balance was assessed using the one-legged stance test (OLST). Mobility was evaluated using Timed Up and Go Test (TUG) time and 5 m walking time. Mental function was evaluated using the Trail-Making Test-Part A (TMT-A) and the MMSE. Each measurement item was measured in a large public facility, with separate spaces designated for each item.

The CS-30 was performed in accordance with the protocol described by Nakatani et al. [16]. Subjects began the test seated upright in a chair that did not have armrests (height: 42 cm), with their arms folded across their chest. Before beginning the test, a researcher gave three verbal instructions: “Please keep your arms crossed over your chest,” “Fully straighten your knees when you stand up,” and “Please continuously repeat the exercise as quickly as you can.” Subjects were video-recorded as they performed the test: standing fully upright and returning to the initial seated position as many times as they could within 30 s. The number of sit–stand–sit cycles completed in 30 s was recorded; cycles not completed by the 30 s mark were not included in this total count (“CS-30 score”).

In addition, cycle counts were separately determined for the first, s, and third 10 s segments of the test (T1: 1–10 s, T2: 11–20 s, T3: 21–30 s). Similarly, cycles not completed by the end of a 10 s segment were not included in the corresponding count. For analysis, subjects were classified into four subtypes based on the numbers of sit–stand–sit cycles per segment, as follows:

1. Steady-goers: Cycle count remained constant across all segments (T1 = T2 = T3).
2. Fluctuators: Cycle count varied among the three segments, either rising and then falling (T1 < T2 > T3), or falling and then rising (T1 > T2 < T3).
3. Decelerators: Cycle count decreased over successive segments (T1 > T2 > T3; or T1 > T2 = T3; or T1 = T2 > T3).
4. Accelerators: Cycle count increased over successive segments (T1 < T2 < T3; or T1 < T2 = T3; or T1 = T2 < T3).

Trunk function was assessed through performance in a sit-up test, which was conducted following the method described by Abe et al. [18]. Before commencing the test, subjects lay in the supine position with both arms folded over their chest, knees bent at 90°, and feet together on the ground; their ankles were held by a technician. One sit-up was defined as lifting the upper torso until the elbows touched the thighs, and then returning to the initial position. The number of repetitions completed in 30 s was counted. Beforehand, each subject was asked to perform a practice sit-up. If they were unable to do so, they did not proceed to the actual test: for these individuals, their performance was simply recorded as zero repetitions.

Knee-extension strength was measured using the method described by Bohannon [19]. Subjects began in a seated position, with hips and knees flexed at 90°. They were instructed to perform an isometric contraction (of the quadriceps) of one leg at maximal effort, pressing the lower shin, near the ankle, against the sensor pad of a handheld dynamometer (μTasF-1, Anima Corporation, Tokyo, Japan). Four measurements were obtained in total, two each for the left and right leg; the maximum observed measurement was selected for analysis.

Toe-grip strength was measured using the method described by Souma et al. [20]. Subjects began in a seated position, with knees and hips flexed at 90°. One foot was placed over a toe-grip dynamometer (TKK 3362, Takei Scientific Instruments, Niigata Prefecture, Japan), with the handle positioned under the first proximal phalanx. Consistent toe position was ensured by sliding the device’s “heel stopper” to match the length of the foot. Subjects were instructed to curl their toe at maximal effort. Four measurements were obtained in total, two each for the left and right foot; the maximum observed was selected for analysis.

Balance was evaluated through OLST time. The test was performed according to the method described by MacRae et al. [21], but modified slightly for static balance. Subjects were instructed to stand with their arms at their sides and to keep both eyes open, and then to lift one foot off the ground and keep it raised for as long as possible (maximum: 120 s). Measurement commenced when their foot left the ground, and ended when the supporting leg moved or the suspended foot touched the ground, or once the maximum time was reached. The OLST was performed twice in total, once per leg: the longest time (of the two trials) was selected for analysis.

Mobility was assessed using two measures: TUG time and 5 m walking time. The TUG was performed using the method described by Podsiadlo et al. [22]. Subjects began the test seated upright in a chair without armrests (height: 42 cm). They were instructed to, once given the start signal, stand up from the chair, walk to a point 3 m away and back as quickly as possible, and then sit back down in the chair. The time taken to complete this task was recorded for analysis.

In addition, 5 m walking speed was assessed using the method described by Amano et al. [23]. Subjects were instructed to walk 11 m in a straight line on level ground as quickly as possible. To eliminate potential confounders induced by acceleration near the start of the test and deceleration near the end, walking time was only measured for the middle 5 m segment (i.e., ignoring the first and last 3 m segments).

Attentional function was assessed using the TMT-A [24]. Subjects were presented with a sheet of paper on which the numerals 1–25 were randomly distributed. They were instructed to draw lines to connect the numbers in ascending order. Performance was assessed in terms of the time required to complete the task (i.e., connect all of the numbers).

Global cognitive function was evaluated using the MMSE [25]. The MMSE is a short test that is widely used internationally to efficiently assess cognitive function: it comprises 11 items and tasks such as writing letters and reproducing shapes. Performance was assessed in terms of total MMSE score across all items.

Statistical analysis was performed as follows. First, data normality was confirmed for each attribute and assessment item using the Shapiro–Wilk test. CS-30 score (total cycles) was tested for gender differences using the Mann–Whitney U test. Each assessment item was tested for differences in terms of the four CS-30 patterns using the Kruskal–Wallis test (with Bonferroni correction). Finally, CS-30 patterns were tested for associations with each assessment item using Spearman rank-order correlation coefficients. SPSS Statistics software (v.24: IBM, SPSS Inc., Chicago, IL, USA) was used for all analysis; the significance level was set at 5%.

## 3. Results

Considering the sample as a whole, the mean CS-30 score was 22.4 ± 6.0 sit–stand–sit cycles. No significant difference was observed between genders (men: 21.7 ± 6.0 vs. women: 22.6 ± 6.0, *p* = 0.561).

Subjects were grouped as follows, based on their relative performance across the three 10 s segments: steady-goers: *n* = 77 (38%), fluctuators: *n* = 31 (15%), decelerators: *n* = 65 (32%), accelerators: *n* = 29 (15%; Table 2). Basic attributes and all assessment items were compared between these four groups. CS-30 score was significantly higher among accelerators than steady-goers (*p* < 0.05). Segmental cycle (sub)counts were also compared between the four groups. During T1, decelerators and accelerators completed significantly more cycles than steady-goers (*p* < 0.05); during T2 and T3, accelerators completed significantly more cycles than steady-goers and decelerators (*p* < 0.05). Significantly stronger knee extension was measured for accelerators than steady-goers and decelerators (*p* < 0.05); however, no other significant differences between the four groups were observed for any of the other assessment items.

CS-30 score (total cycles) was tested for associations with basic attributes and each assessment item. Significant correlations with mobility—both in terms of TUG time and 5 m walking time—were consequently observed in each of the four groups (Table 3). Significant correlations with age, each muscle function item, and balance were observed among steady-goers and fluctuators. Other notable correlates with CS-30 score included TMT-A time (for steady-goers and decelerators), sit-up count (decelerators), and MMSE score (accelerators).

## 4. Discussion

In this study, we hypothesized that different patterns of CS-30 performance evince the physical and mental characteristics of community-dwelling older adults in Japan. To verify this hypothesis, we defined the categories of CS-30 performance among healthy community-dwelling older Japanese adults—namely, in terms of how sit–stand–sit cycle rates vary over successive segments of the test. We then sought to determine the distributions of group attributes and compare the indicators of mental and physical function across the three variant groups (fluctuators, accelerators, and decelerators) with respect to the reference group (steady-goers). Our research yielded several major findings. First, the two most common temporal patterns of CS-30 performance—steady-goers and decelerators, respectively—accounted for 70% of the study population. Second, accelerators completed significantly more sit–stand–sit cycles in total than steady-goers, and exhibited significantly stronger knee extension than both steady-goers and decelerators. Finally, correlation analysis revealed significant differences between these patterns in terms of several indicators of physical and mental/cognitive functions: predominantly among steady-goers and fluctuators, CS-30 score (total cycles) was significantly associated with measures of muscle function, balance, and age; meanwhile, CS-30 score was moderately correlated with MMSE score among accelerators only.

Normative (average) scores for CS-30 performance have previously been published for community-dwelling older adults in Japan: for men/women aged 65–69 years, they are 16–20/17–21 cycles, respectively; for those aged 70–74: 16–20/15–19 cycles, respectively; for those aged 75–79: 15–17/13–17 cycles, respectively, and for those aged 80+: 14–16/13–16 cycles, respectively [16]. These normative scores show that our subjects’ CS-30 scores tended to be slightly higher than the average for their corresponding age group. A possible explanation for our participants’ greater physical capacity when compared to typical community-dwelling older adults is high health consciousness, as all of our participants voluntarily agreed to participate in the panel of fitness tests. In addition, we observed no significant gender differences in CS-30 score. Many preceding studies involving the CS-30 have investigated whether performance varies between men and women, but all found that gender-related differences tend to diminish with age [15,16,26]. We speculate that the negligible gender difference we observed regarding CS-30 score was primarily a product of the advanced age of our study population, as most of our subjects were in their 70s.

Our analysis groups were defined based on the temporal changes in sit–stand–sit (sub)count over three equal (10 s) segments of the CS-30. As mentioned, the two most common patterns were steady-goers and decelerators. One aspect common to both patterns was the lack of gradual acceleration over successive segments; cycle count either remained constant (steady-goers) or gradually reduced (decelerators) over time. Greater cycle counts in later segments suggest that the subject in question was not moving at their maximum capacity in earlier segments. Notably, our subjects had well-preserved cognitive and physical function; thus, it can be assumed that most subjects correctly understood the test instructions and, therefore, continued to exert maximal effort throughout the task. Based on these considerations, we conclude that two temporal patterns of CS-30 performance predominate among healthy community-dwelling older adults: steady-goers, who are characterized by invariance in the sit–stand–sit cycle rate over time; and decelerators, who are characterized by a gradual reduction in this rate.

Steady-goers exhibited significant correlations of CS-30 score with age, sit-up repetitions, quadriceps strength, toe-grip strength, OLST time, TUG time, 5 m walking time, and TMT-A score. These observations corroborate previous reports of relationships between CS-30 score and these correlates in the context of community-dwelling older adults (age: [11], sit-up repetitions: [11], knee-extension strength: [12,16], toe-grip strength: [27], OLST time: [11], TUG time: [12], walking speed: [12], TMT-A score: [11]). We suspect that the constant rate of sit–stand–sit repetitions exhibited by our steady-goers is the most typical pattern for CS-30 performance among general community-dwelling older adults.

Fluctuators showed significant correlations of CS-30 score with nearly all of the assessment items found for steady-goers; TMT-A score was the lone exception. This indicates that individuals of both subtypes have similar physical functional capacity; this hypothesis is strengthened by the fact that no significant differences were observed between steady-goers and fluctuators for any assessment item. We conclude that older adults who exhibit both acceleration and deceleration during the CS-30 share similar attributes to their “normal” counterparts who perform the task at a constant rate.

Decelerators completed significantly more sit–stand–sit cycles than steady-goers during the T1 segment; however, no such difference was observed for T2 or T3. CS-30 performance reportedly reflects muscular endurance [10]. Thus, we suppose that our decelerators had poorer muscular endurance than our steady-goers, since individuals with low (muscular) endurance would be expected to complete fewer cycles as the test continues. Furthermore, while some of the correlates with CS-30 score identified among steady-goers were not observed among decelerators—namely, knee-extension strength and toe-grip strength—significant associations were observed with sit-up repetitions and TMT-A score. As with the CS-30, sit-ups are an exercise that requires muscular endurance [28]. In addition, the ability to sustain one’s attention would seem to be necessary to repeatedly and continuously activate the muscle groups involved for the duration of a 30 s task [14]. We believe these two connections explain why CS-30 score was linked with measures of muscular endurance and attentional function among decelerators. Our findings suggest that community-dwelling older adults who are classified as decelerators based on CS-30 performance require training that focuses on improving their muscular endurance.

Accelerators showed significantly greater CS-30 score and knee-extension strength when compared to steady-goers. The CS-30 is designed to assess lower-limb function in an integrated manner [15]; performance in such tasks has been reported to be associated with activities of daily living (ADL) ability [29]. In addition, knee-extension weakness is a reported predictor of ADL and mobility-related disability [30]. For these reasons, we believe that the accelerators in our study generally possessed greater physical function than the steady-goers. One previous study observed that, while healthy, younger adults tend to underestimate their own physical capacity, frail, older adults tend to overestimate it [31]. Our groups were comparable in terms of age: nevertheless, it is possible that our accelerators—a subtype with apparently high physical capacity—underestimated their physical capacity, at least at the start of the CS-30. If true, this subtype may (unintentionally) not have utilized their maximum potential at the very start of the task, resulting in fewer sit–stand–sit cycles early in the trial, but then gradually accelerated their speed during the second half. In addition, uniquely among the four patterns, accelerators exhibited a significant correlation between CS-30 score and MMSE score. This finding is consistent with a previous observation that community-dwelling older adults with higher cognitive function tend to complete more sit–stand–sit cycles in the CS-30 [13]. It is unclear why this trend was not observed in the other groups; perhaps it was a consequence of the rather narrow distribution of MMSE scores overall, as the cognitive function of our study population was relatively intact. Therefore, it is possible that, among accelerators, completing fewer sit–stand–sit cycles in the CS-30 is a sign of the preclinical stage of dementia [32], in which case, additional assessments and interventions targeting mental functioning would likely be needed.

Our study has several limitations. The first was the small number of study participants, especially fluctuators and accelerators. Subsequent studies with a larger sample size are required to confirm our findings. Second, our subjects were sourced from a population of older adults who were still in relatively good health. In the future, it will be necessary to determine whether similar results are found in frail older populations. Third, our cross-sectional design prevented us from inferring causality for any of the associations identified. Future longitudinal studies are needed to clarify the relationship between changes in each function and temporal patterns in the performance of CS-30. These limitations notwithstanding, we believe our findings provide valuable information for healthcare professionals, and can help them design intervention programs for older adults.

In conclusion, we determined that differences in physical and mental characteristics are predicted by specific temporal patterns of CS-30 performance in a Japanese population of community-dwelling older adults. Our findings suggest that geriatric experts should customize intervention programs for individuals based on the specific pattern of variation these patients show over the course of the task. For example, fluctuators and steady-goers may benefit most from typical interventions designed to prevent the need for long-term care in normal older adults, while interventions for decelerators should aim to improve muscle endurance. In addition, accelerators who score poorly on the test overall (in terms of total cycles) should be monitored for early-stage cognitive decline. The method presented in this study is expected to improve the operational efficiency of busy healthcare workers due to its ability to obtain more information from a single indicator. Despite some limitations, we believe that our findings will be useful for healthcare professionals who are involved in assessing the physical and mental capabilities of older adults, as well as those who determine goals for associated intervention programs.

## Figures and Tables

**Table 1:** Demographic characteristics of the 202 subjects.

Variable	Data
Age (year)	73.3 ± 6.0
Sex: Men/Women (n)	42/160
Height (cm)	154.8 ± 7.6
Weight (kg)	53.3 ± 8.3
BMI (kg/m^2^)	22.2 ± 2.6

Data are presented as mean ± standard deviation. BMI: Body Mass Index.

**Table 2:** Comparisons of the basic attributes and assessment items for each 30 s chair-stand test (CS-30) pattern.

Variable		Steady-Goers	Fluctuators	Decelerators	Accelerators	χ^2^	*p*
Attribute	Age (year)	73.6 ± 5.8	75.0 ± 7.8	73.9 ± 5.4	72.1 ± 5.6	5.47	0.14
	Height (cm)	154.3 ± 7.8	154.6 ± 8.5	155.1 ± 6.8	155.3 ± 7.7	0.44	0.93
	Weight (kg)	53.0 ± 8.5	51.6 ± 9.1	54.0 ± 7.6	54.4 ± 8.4	3.50	0.32
CS-30	Score (cycles)	20.7 ± 5.0	23.2 ± 6.8	22.5 ± 5.8	25.8 ± 6.8 *	14.51	<0.01
	T1 (cycles)	7.0 ± 1.8	7.9 ± 2.1	8.1 ± 1.9 ^†^	8.1 ± 2.2 *	13.28	<0.01
	T2 (cycles)	7.0 ± 1.8	8.3 ± 2.3	7.4 ± 1.9	8.8 ± 2.2 * ^‡^	17.40	<0.01
	T3 (cycles)	7.0 ± 1.8	7.8 ± 2.2	6.9 ± 1.8	9.3 ± 2.4 * ^‡^	26.57	<0.01
Muscle function	Sit-up (repetitions)	8.2 ± 7.1	10.6 ± 6.7	9.4 ± 6.4	10.2 ± 8.2	3.71	0.29
	Knee-extension strength (kg)	22.2 ± 7.2	22.7 ± 5.5	21.7 ± 4.9	26.6 ± 8.6 * ^‡^	8.52	0.04
	Toe-grip strength (kg)	8.5 ± 4.5	9.1 ± 4.4	9.2 ± 3.3	9.9 ± 4.6	2.59	0.46
Balance	OLST time (s)	41.8 ± 39.7	40.3 ± 38.1	41.2 ± 38.0	53.6 ± 37.2	3.58	0.31
Mobility	TUG time (s)	5.8 ± 1.2	5.8 ± 1.1	5.5 ± 1.1	5.2 ± 0.9	8.54	0.04
	5 m walking time (s)	2.8 ± 0.6	2.8 ± 0.4	2.7 ± 0.5	2.6 ± 0.5	4.13	0.25
Mental function	TMT-A score (s)	104.2 ± 34.3	109.0 ± 36.0	101.6 ± 26.2	95.1 ± 28.2	2.75	0.43
	MMSE score (pts)	28.5 ± 2.1	28.3 ± 1.7	28.2 ± 3.0	28.9 ± 1.7	2.84	0.42

Data are presented as mean ± standard deviation *Accelerators > Steady-goers, ^†^: Decelerators > Steady-goers, ^‡^ Accelerators > Decelerators. Kruskal–Wallis test, with post-hoc Bonferroni correction. T1: cycle count for the first 10 s (of the CS-30), T2: cycle count for the second 10 s, T3: cycle count for the third 10 s. CS-30: 30 s chair-stand test, OLST: one-legged stance test, TUG: timed up and go test, TMT-A: Trail-Making Test-Part A, MMSE: Mini-Mental State Examination.

**Table 3:** Correlations of CS-30 performance (total cycles) with assessment items.

Variable		Steady-Goers		Fluctuators		Decelerators		Accelerators	
Attribute	Age (year)	−0.36	**	−0.45	*	−0.11		−0.18	
	Height (cm)	−0.08		0.10		0.12		−0.23	
	Weight (kg)	−0.13		0.06		0.04		−0.33	
Muscle function	Sit-up (repetitions)	0.38	**	0.55	**	0.27	*	0.36	
	Knee-extension strength (kg)	0.31	**	0.40	*	0.18		0.15	
	Toe-grip strength (kg)	0.25	*	0.36	*	0.22		0.09	
Balance	OLST time (s)	0.47	**	0.66	**	0.20		0.12	
Mobility	TUG time (s)	−0.60	**	−0.54	**	−0.55	**	−0.64	**
	5 m walking time (s)	−0.47	**	−0.56	**	−0.50	**	−0.49	**
Mental function	TMT-A score (s)	−0.29	*	−0.22		−0.30	*	−0.18	
	MMSE score (pts)	0.17	**	−0.01	*	0.24		0.49	**

Data presented are Spearman rank-order correlation coefficients. *: *p* < 0.05, **: *p* < 0.01. CS-30: 30-s chair-stand test, OLST: one-legged stance test, TUG: timed up & go test, TMT-A: Trail-Making Test-Part A, MMSE: Mini-Mental State Examination.
